# Association of gestational age with the option of pregnancy termination for fetal abnormalities incompatible with neonatal survival

**DOI:** 10.1590/S1679-45082016AO3721

**Published:** 2016

**Authors:** Flavia Westphal, Suzete Maria Fustinoni, Vânia Lopes Pinto, Patrícia de Souza Melo, Anelise Riedel Abrahão

**Affiliations:** 1 Universidade Federal de São Paulo, São Paulo, SP, Brazil

**Keywords:** Congenital abnormalities, Fetal viability, Abortion, legal, Decision making

## Abstract

**Objective:**

To identify the profile of women seen in a Fetal Medicine unit, diagnosed with fetal abnormality incompatible with neonatal survival in their current pregnancy, and to check the association of gestational age upon diagnosis with the option of pregnancy termination.

**Methods:**

This is a retrospective cohort study carried out in the Fetal Medicine Outpatients Clinic of a university hospital, in the city of São Paulo (SP), Brazil, using medical records of pregnant women with fetus presenting abnormalities incompatible with neonatal survival. The sample comprised 94 medical records. The Statistical Package for the Social Sciences (SPSS), version 19, was used for the data statistical analysis.

**Results:**

The population of the study included young adult women, who had complete or incomplete high school education, employed, with family income of one to three minimum wages, single, nonsmokers, who did not drink alcoholic beverages or used illicit drugs. Women with more advanced gestational age upon fetal diagnosis (p=0.0066) and/or upon admission to the specialized unit (p=0.0018) presented a lower percentage of termination of pregnancy.

**Conclusion:**

Due to characteristics different from those classically considered as of high gestational risk, these women might not be easily identified during the classification of gestational risk, what may contribute to a late diagnosis of fetal diseases. Early diagnosis enables access to specialized multiprofessional care in the proper time for couple's counseling on the possibility of requesting legal authorization for pregnancy termination.

## INTRODUCTION

Congenital anomalies (CA), birth defects, congenital disorders, and congenital malformations are synonyms used to describe developmental disorders present at birth. They are consequences of failure of one or more body parts during the embryonic development; they can be structural, functional, metabolic, behavioral or hereditary.^(1)^ The defects may be present alone or in combination, comprising syndromes.

The etiology of CA may be related to genetic, environmental or multifactorial factors or remain unknown. It is estimated that 40 to 60% of all CAs have unknown cause. Among the known causes, the most common are multifactorial, related to combinations of genetic and environmental factors.^(1,2)^

The overall incidence of birth defects in Brazil is between 2 and 5% of all live births, not different from that found in other regions of the world.^(3)^

Mortality from CA affects children mainly in the first year of life. With the reduction of other causes of child mortality, such as diarrhea and respiratory infections, the percentage of deaths from malformations has become proportionally higher. In Brazil, CA rank second in cause of death of children.^(4)^

Congenital anomalies have great impact on the family and generate feelings of sadness for the loss of the idealized child, fear of the unknown, fear of difficulties to be faced, and a high risk of family breakdown.^(2,5)^

The diagnosis of a structural abnormality in the fetus enables antenatal counseling and planning of actions to be taken in the postnatal period.^(6)^

In order to improve the diagnostic approach of CA, there has been a revolution in recent decades in the field of diagnostic imaging in obstetrics, providing images of the developing fetus, evaluation of growth and behavior, and increasingly earlier recognition of the abnormalities. Some examples of these technologies are two-dimensional and three-dimensional ultrasound, echocardiography, Doppler flowmetry and magnetic resonance, among otehrs.^(7)^

Along with the biotechnological evolution of fetal assessment, there was a breakthrough in genetics, with the development of molecular biology and the Human Genome Project, in which many diseases previously evaluated only in terms of statistical risk of occurrence and/or recurrence, are now diagnosed in-utero.^(8)^

Once the fetal diagnosis is established, in some cases, it is possible to propose alternative in-utero treatment, enabling better prognosis. However, for some fetal abnormalities that will lead to fetal death, the parents should be informed about the high risk of intrauterine, neonatal or infant death. Lethal fetal diseases, also described as nonviable or incompatible with neonatal survival, invariably lead to intrauterine fetal death or death in the neonatal period, regardless of attempting supportive measures or treatment.^(5,9)^

In these circumstances, in many parts of the world, such diagnoses are often followed by the couple deciding for abortion.^(5,6)^

In developed Western countries in Europe and North America, the termination of pregnancies in which the fetus has an anomaly incompatible with life is legal.^(10,11)^

In Brazil, the law only supports voluntary interruption of pregnancy in cases where the woman was victim of sexual violence, when the pregnancy is life-threatening for the mother,^(4)^ and, more recently, in the event of an unequivocal diagnosis of anencephaly.^(12)^

Women have therefore the legal right to decide whether to take forward or interrupt a pregnancy of an anencephalic fetus; nevertheless, this is not the only fetal anomaly incompatible with neonatal survival; there remains the concern about the outcome of other pregnancies, for which legal authorization is still required.

In face of a diagnosis of nonviable fetus, social and cultural aspects may interfere in the couple's decision. Therefore, specialized care and time for decision-making are key factors to prevent future mental disorders.^(13)^ Furthermore, maternal risks involved in continuing pregnancy should be considered and discussed with the parents, thus helping in the decision-making process of interrupting or not pregnancy.^(6)^

## OBJECTIVE

To identify the profile of pregnant women with fetus presenting anomalies incompatible with neonatal survival, and to check whether gestational age influenced in the decision to request pregnancy interruption.

## METHODS

A retrospective cohort study, conducted at the Fetal Medicine Outpatients Clinic – *Universidade Federal de São Paulo* (UNIFESP). Data collection was initiated after approval by the Internal Review Board of UNIFESP, protocol number 507.887, CAAE: 19093813.0.0000.5505. The coordinator signed the Terms of Data Collection Authorization for the Fetal Medicine Outpatients Clinic of UNIFESP.

The population consisted of all records of women seen at the Fetal Medicine Outpatients Clinic – UNIFESP, from January 1^st^, 2010 to December 31^st^, 2013, who were diagnosed with fetal anomaly incompatible with neonatal survival in the current pregnancy, at any gestational age.

The fetuses were identified as unviable after evaluation by a team of specialized in fetal Medicine Physicians. Lethality was assumed in the presence of an anomaly or set of anomalies that would result in a likely intrauterine death, or if born live, in failure to independently survive without life support.

In cases established as nonviable, it was possible to request a court order for legal voluntary termination of pregnancy, when the couple so preferred. As from May 10, 2012, resolution 1989 provided for the legal termination of pregnancy in cases of anencephaly.^(12)^

The inclusion criterion was that the woman had received antenatal care of this pregnancy in this Outpatients Clinic until the outcome. Women were excluded if they were not enrolled, did not require hospitalization or those who did not undergo interruption or delivery in this unit.

Of the initial sample of 166 women with fetuses with lethal CA seen in our service, during the study period, 49 were excluded due to lack of records related to hospital admission for termination of pregnancy/delivery; 14 because the records were not provided by the Medical Records and Statistics Service (SAME – *Serviço de Arquivo Médico e Estatística*), and 9 for not having registry in the Clinic. The final sample consisted of 94 medical records of women who received a diagnosis of fetal anomalies incompatible with neonatal survival in the current pregnancy.

The software used for data analysis was the Statistical Package for the Social Sciences (SPSS) version 19. Initially, we performed a descriptive study of the variables in the database. Continuous variables were analyzed by calculating mean, median, standard deviation, minimum and maximum. For categorical variables, frequency and percentage were calculated.

In order to compare categorical variables for interruption or continuation, we used the χ^2^ test and, whenever necessary, the odds ratio.

All analyzes were planned and developed taking into account a significance level of 5% (p<0.05).

## RESULTS

The sociodemographic characteristics are depicted on table 1, emphasizing the most frequent categories.

**Table 1:** Distribution of women studied, according to sociodemographic data and habits

Characteristics	Total (n=94)
	n (%)
Age in years, mean ± standard deviation	28.46±7.15
White skin	49 (52.1)
Born in the Southeastern Region	60 (63.8)
Complete/incomplete High School	51 (54.3)
Catholic	57 (60.6)
Employed	56 (59.6)
Single	55 (58.5)
Lives with partner	76 (80.9)
Family income between 1 and 3 minimum wages	48 (51.1)
No smoking	82 (87.2)
No alcohol drinking	67 (71.3)
Never used illicit drugs	83 (88.3)

Of the records analyzed, 33% had one or more chronic diseases (n=31), the more prevalent being anemia (22.6%), and hypertension (19.4%).

In 67% (n=63) of the records, registries on use of medication were found, in isolation or in combination, the more prevalent being vitamins (46%), followed by analgesic agents (23.8%), antibiotics (15.9%), antihypertensive drugs (7.9%), and antiemetic drugs (1.6%).

In regards to obstetric history, the records indicated that women had a mean of 2.31 pregnancies and most of them had not planned the current pregnancy (73.4%), as shown on table 2.

**Table 2:** Distribution of women studied, according to obstetric history

Characteristics	Total (n=94)
	Mean ± standard deviation
Pregnancies	2.31 (1.38)
Parity	0.88 (1.14)
Abortions	0.45 (0.68)
Live births	0.83 (1.14)
Current pregnancy not planed	69 (73.4)
No fetus malformations in previous pregnancies	89 (94.7)

Most patients received fetal diagnosis (79.8%) and/or came to the specialized clinic (85.1%) only in the second trimester (from 14 weeks 1/7 to 28 weeks). Only 8.5% (n=8) of women were diagnosed with fetal anomalies incompatible with neonatal survival during the first trimester, as displayed on table 3.

**Table 3:** Distribution of women studied, according to characteristics related to fetal diagnosis

Characteristics		Total (n=94)
		n (%)
Fetal disorder		
	Central nervous system	36 (38.3)
	Genitourinary system	20 (21.3)
	Chromosomal abnormalities	18 (19.1)
	Thoraco-abdominal closing defect	9 (9.6)
	Multiple malformations	8 (8.5)
	Musculoskeletal system	3 (3.2)
Gestational age upon fetal diagnosis		
	Up to 14 weeks	8 (8.5)
	14 weeks 1/7 to 21 weeks	48 (51.1)
	21 weeks 1/7 to 28 weeks	27 (28.7)
	More than 28 weeks	11 (11.7)
Gestational age upon first visit to the clinic		
	Up to 14 weeks	3 (3.2)
	14 weeks 1/7 to 21 weeks	41 (43.6)
	21 weeks 1/7 to 28 weeks	39 (41.5)
	More than 28 weeks	11 (11.7)

Most women had no consanguineous partner (93.6%) and no family history of CA (85.1%).

Of the medical records studied, gestational interruption was conducted in 41 (43.6%) cases and maintenance of pregnancy in 53 (56.4%).

The more advanced the gestational age upon fetal diagnosis (p=0.0066) and/or first visit to the specialized clinic (p=0.0018), the lower the rate of request to interrupt pregnancy, as can be seen on table 4.

**Table 4:** Characteristics related to fetal diagnosis of women studied, according to the choice of interrupting pregnancy

Variables		Requested interruption			p value
		Yes	No	Total	
		n (%)	n (%)	n (%)	
Gestational age upon fetal diagnosis					
	Up to 14 weeks	6 (75)	2 (25)	8 (100)	0.0066*
	14 weeks 1/7 to 21 weeks	26 (54.2)	22 (45.8)	48 (100)	
	21 weeks 1/7 to 28 to weeks	7 (25.9)	20 (74.1)	27 (100)	
	More than 28 weeks	2 (18.2)	9 (81.8)	11 (100)	
Total		41 (43.6)	53 (56.4)	94 (100)	
Gestational age upon first visit to the clinic					
	Up to 21 weeks	27 (61.4)	17 (38.6)	44 (100)	0.0018**
	21 weeks 1/7 to 28 weeks	13 (33.3)	26 (66.7)	39 (100)	
	More than 28 weeks	1 (9.1)	10 (90.9)	11 (100)	
Total		41 (43.6)	53 (56.4)	94 (100)	

* odds ratio;

** χ^2^ test.

## DISCUSSION

This study comprised a population of young adult pregnant women with schooling level of complete/incomplete High School, employed, with family income between one and three minimum wages, single but living with a partner. More than half of them were born in the Southeastern region (63.8%), as expected, since the clinic is located in the city of São Paulo.

The mean age was 28.46 years, or less, younger than the age classically considered at risk for development of fetal anomalies. Another study conducted in São Paulo with pregnant women and fetuses with lethal malformation found similar results, with a mean age of 25 years.^(14)^

Most of the study population did not use tobacco (87.2%), alcoholic beverages (71.3%) or illicit drugs (88.3%). These data, contrary to expected, revealed a population with different characteristics from those considered as high-risk pregnancy, and similar to those of pregnant women in general.^(15)^ Thus, it is noteworthy the fact that these women probably started their antenatal care at a low risk service, and were not easily identified during the gestational risk classification, which may have contributed to late diagnosis of fetal disease.

In this study, alcohol was used by a minority of pregnant women, but its teratogenic potential was highlighted. Alcohol intake during pregnancy has been identified for decades as responsible for the occurrence of fetal abnormalities, and the current consensus is that there is no safe limit of intake during pregnancy.^(16)^

Alcohol is a known teratogen that poses a severe risk for development of the fetal central nervous system. Its chronic consumption in high doses is associated with fetal alcohol syndrome, but moderate use also has some effects, including permanent neurobehavioral disorders.^(17)^

Although the population includes young adult pregnant women, 33% of them had chronic diseases, and anemia and hypertension were the most frequently reported. Moreover, 67% (n=63) of pregnant women used medications. Maternal anemia is associated with increased rates of maternal and fetal mortality and infectious diseases, as well as increased risk of prematurity and low birth weight. Furthermore, one of the causes may be folate deficiency, which is associated with increased risk of neural tube closing defects during pregnancy.^(18)^

As for hypertension in pregnancy, if medication is used, the adequacy of the previously used drugs should be taken into account, since some antihypertensive agents, such as angiotensin-converting enzyme inhibitors and angiotensin receptor blockers, are contraindicated because of their teratogenic potential.^(15,17)^

The teratogenic potential of exposing pregnant women to certain medications is already known and has been widely studied. In 1982, the Food and Drug Administration (FDA) established five categories (A, B, C, D and X) to indicate the teratogenic potential of each drug.^(19)^

In this study, the most prevalent drug class was vitamins. Despite being considered a specific medication by the Brazilian National Health Surveillance Agency (ANVISA),^(20)^ vitamins are classified as category A, according to the risk of teratogenicity proposed by the FDA, that is, they do not have teratogenic potential. However, excluding the use of vitamins, we found exposure to drugs of other therapeutic classes in 53.2% (n=50) of the women studied.

In a study in the city of Maringa (PR), with records of malformed fetuses, exposure to drugs during pregnancy was observed in 63.2% of records. Of these, 51.5% were drugs classified in the categories B, C, and D, as proposed by the FDA, and 12.1% in Category X, considered of high teratogenic risk. This study concluded that the occurrence of CA may be related to substance use during pregnancy, given the high percentage of drugs used with high teratogenic risk.^(21)^

Considering the problem of alcohol, tobacco, and medication consumption by women, the Ministry of Health established some specific actions to be taken on the preconception phase, as described on the Manual for Low Risk Antenatal Care, published in 2012. However, very few women seek this care, since most pregnancies are unplanned.^(15)^

Gestational planning, or lack of it, is worth analyzing, since in unplanned pregnancy the woman may have contact with teratogenic agents without concern, because the possibility of pregnancy is not considered.

In this study, we observed most women (73.4%) had not planned the current pregnancy, a fact confirmed by other investigations, which found that the majority of the population that seeks public antenatal services does not plan pregnancy.^(22)^

Furthermore, due to non-gestational planning, very few women have the opportunity of receiving a preconception evaluation to investigate current and previous health problems that may interfere in the normal progress of a future pregnancy. The possibility of changing treatment to medications with less teratogenic potential and better clinical control of existing chronic diseases should also be considered, providing better maternal and perinatal outcomes.^(15)^

In relation to gestational age at the first visit to the clinic, according to the medical records most women were admitted in the second trimester; 43.6% with a gestational age of 14 1/7 weeks to 21 weeks, and 41.5% with gestational age of 21 weeks 1/7 to 28 weeks. Most often, it was at this moment that the antenatal diagnosis of fetal CA incompatible with neonatal survival was made. We observed that only a small portion of pregnant women received a diagnosis of fetal anomalies incompatible with neonatal survival during the first trimester, and the percentage of those attending the reference service in that period was even lower (3.2%). This scenario is similar to those found in a study carried out in the same department, with data from women seen between 2000 to 2006, where the diagnosis of fetal anomaly incompatible with neonatal survival was made up to the 13^th^ week in only 17% of pregnancies.^(9)^

Although the Ministry of Health recommends only one obstetric ultrasound during pregnancy, and does not consider it a mandatory exam for low-risk pregnancies,^(15)^ it is known that this exam is a critical tool in the diagnosis of developmental anomalies for screening fetal morphology, placenta and amniotic fluid.^(23)^

A study conducted with pregnant women seen in primary care in the city of Goiania (GO) showed that only 41.8% of them had one obstetric ultrasound per trimester of pregnancy.^(24)^ A national hospital-based investigation, including postpartum women and newborns, demonstrated that although the majority of women (98.2%) referred having had an antenatal ultrasound, the report was available only in part of the prenatal cards analyzed (62.8%). From the records available, we found that only a quarter of the exams was performed before the 14^th^ gestational week, when it is better to calculate gestational age. This calculus is essential to monitor the course of pregnancy and any decisions related to its termination.^(25)^

The results showed that the more advanced the gestational age upon diagnosis (p=0.0066), and the gestational age on first visit to the clinic (p=0.0018), the lower the percentage of request for termination of pregnancy.

We therefore noted that pregnant women faced difficulties to have access to diagnosis of fetal anomaly, as well as to be referred to the specialized clinic. As a consequence, part of them received late specialized care and definite diagnosis of fetal malformation.

A study on factors that influenced the decisions of maintaining pregnancy after diagnosis of a lethal perinatal condition reported the gestational age upon diagnosis did not influence the decision-making.^(26)^ However, study of fetuses with heart disease found that gestational age upon diagnosis was a major factor for the parents to make decision for termination of pregnancy (<0.001).^(27)^

Maternal-fetal attachment increases with gestational age, particularly after experiencing the first fetal movements.^(28)^ This leads us to infer that the more advanced the pregnancy, the greater the involvement of pregnant women with the fetus, and the harder the decision to terminate pregnancy.

## CONCLUSION

The population studied had different characteristics from those considered as high-risk pregnancy. Hence, it is possible that these women will not be easily identified during the gestational risk classification, which may contribute to late diagnosis of fetal diseases. Therefore, all women, regardless of risk factors, should have guaranteed a morphological ultrasound during the first trimester, preferably between the 11^th^ and 13^th^ week of pregnancy.

The more advanced the gestational age upon diagnosis and at the first visit to the clinic, the lower the percentage of requests to interrupt pregnancy.

Early diagnosis of fetal disease provides access to specialized multidisciplinary care in adequate time to counsel the couple about the possibility of judicial authorization request for termination of pregnancy.
